# Ruptured internal carotid artery aneurysm following surgery for chronic sinusitis: delayed presentation: a case report

**DOI:** 10.1093/jscr/rjae010

**Published:** 2024-01-30

**Authors:** Derar Al-Domaidat, Jamal Jawad

**Affiliations:** Otolaryngology Department, Dr. Soliman Fakeeh Hospital, Fakeeh College for Medical Sciences, Clinical Sciences, Jeddah 23323, Saudi Arabia; Otolaryngology Department, Dr. Soliman Fakeeh Hospital, Fakeeh College for Medical Sciences, Clinical Sciences, Jeddah 23323, Saudi Arabia

**Keywords:** petrous aneurysm, internal carotid artery pseudoaneurysm, fungal aneurysm, massive epistaxis, endoscopic sinus surgery, chronic allergic fungal rhinosinusitis

## Abstract

Rupture of internal carotid artery aneurysm has high mortality rate and needs high index of suspicion for immediate management. Massive epistaxis after rupture of aneurysms in the petrous part of internal carotid artery is extremely rare. In this paper, we report the first case of delayed rupture of a petrous carotid aneurysm which developed because of chronic allergic sinusitis.

## Introduction

Aneurysms of the internal carotid artery (ICA) arising from the petrous portion are extremely rare, and most cases are asymptomatic or cause a mass effect leading to palsy of the adjacent cranial nerves. In rupture cases, massive nasal or ear bleeding may lead to death. Fungal aneurysms of ICA are rare. Reported cases are attributed to invasive fungal sinusitis in immunocompromised patients. In this article we report a case of delayed rupture of petrous ICA aneurysm following endoscopic sinus surgery for chronic allergic fungal rhinosinusitis (AFRS).

### Case presentation

A 33-year-old asthmatic female presented with a 1-year history of nasal obstruction, anosmia, frontal headache and blurred vision with no history of epistaxis or previous surgical intervention.

She had bilateral nasal grade 4 polyps with mild proptosis, normal visual acuity and normal cranial nerves examination.

**Figure 1:**
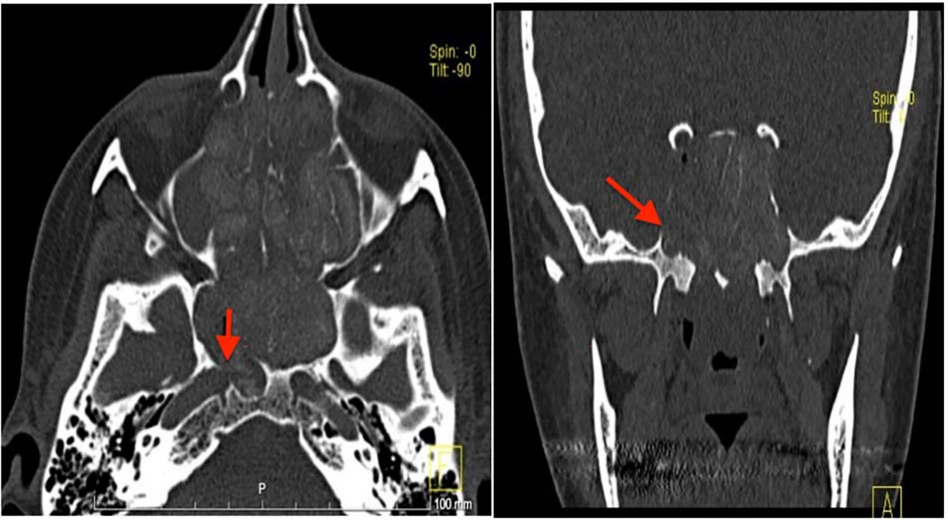
CT scan, left imaging is an axial view, without contrast, showing complete opacification of all paranasal sinuses with heterogeneous density inside the sinuses; with erosion in the distal part of the right petrous carotid canal abutting the right sphenoid sinus (arrow); right image is a coronal view, showing extensive polyposis with bony erosion in the bony boundaries of the sphenoid sinuses.

Computed tomography (CT) scan showed complete bilateral heterogenic opacification of paranasal sinuses, suggestive of chronic AFRS (Fig. 1). More importantly, there was some dehiscence of the lateral wall of the right sphenoid sinus near the petrous segment of the right ICA (Fig. 1). Having no previous history of head injury, trauma or surgical interventions, the existence for any aneurysmal carotid artery was not thought of.

**Figure 2:**
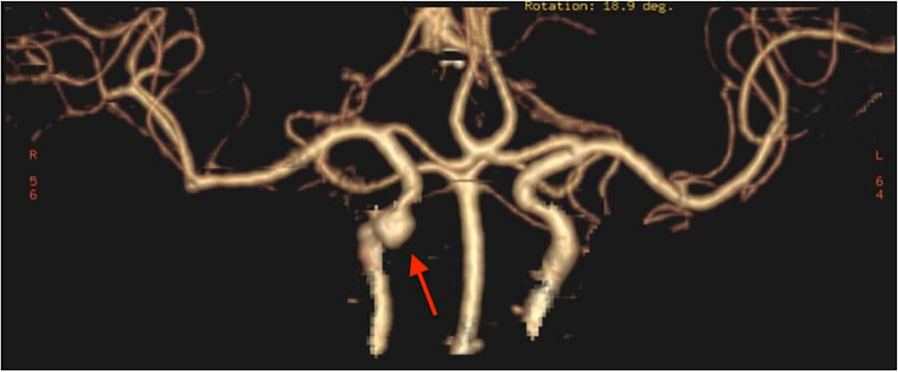
CT angiography, showing pseudoaneurysmal dilatation at the petrous segment of the right ICA.

**Figure 3:**
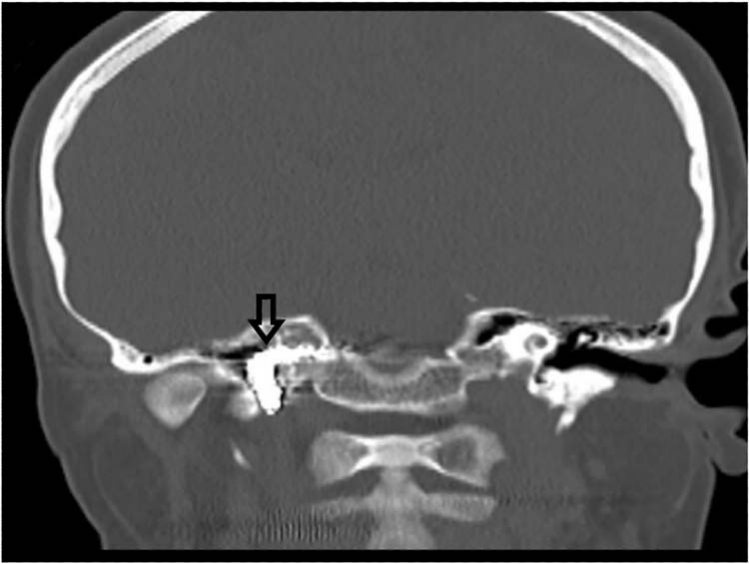
CT scan, showing complete occlusion of the right ICA at the petrous and cervical parts (arrow).

The patient underwent functional endoscopic sinus surgery. Her surgery was uneventful, and all sinuses were opened and cleaned from polyps and fungal debris. During surgery, a non-pulsating bulging at the lower lateral region of the right sphenoid sinus was noticed. This swelling was thought to be a bulging of a dehiscent ICA which may be a normal variant in some patients. Therefore, this bulging mass was not touched and was totally avoided during the procedure. The postoperative recovery period was uneventful and she was discharged the following day. However, on the 13th postoperative day, she presented to the emergency department with severe epistaxis, unconscious and blood pressure was unrecordable. After resuscitation with intravenous fluids and massive blood transfusion with 17 units of packed RBCs, the patient relatively improved and regained consciousness. Anterior and posterior nasal packs were inserted. The CT angiography showed pseudoaneurysm in the petrous segment of the right ICA (Fig. 2). The interventional radiologist performed occlusion with ballooning of right ICA for 45 min without developing motor or cognitive dysfunction, indicating a good compensation by the left ICA. Total occlusion, using multiple coils, was deployed proximal and distal to the aneurysm. Patient was discharged 2 days later. During the follow-up of more than 10 years no neurological deficit was noticed. Recent CT scan showed stable sinus disease and total occlusion of the right ICA (Fig. 3).

## Discussion

The most commonly affected areas of the skull base are the ethmoid, sphenoid, followed by frontal bones. Aneurysms are either true or false (pseudoaneurysm), the difference is the lack of all three layers of the arterial wall in a pseudoaneurysm compared with a true aneurysm, which presents as a dilation of an intact arterial wall [1].

Non-traumatic ICA aneurysms are extremely rare and present a great difficulty in diagnosis, especially if epistaxis is the only symptom. In 2018, Tamada *et al*. [2] reported only 16 cases of ICA aneurysms arising from the petrous portion causing epistaxis.

Etiologies for petrous aneurysms that have been reported include congenital, radiation injury, trauma and infection. The reported infectious causes of petrous aneurysms are deep neck infections, tuberculosis, chronic otitis media and skull base osteomyelitis [3–6]. Another reported infectious cause is invasive fungal sinusitis (aspergillosis and mucormycosis) involving the sphenoid sinus [7–9].

In our case, we think that the chronic inflammatory process and mass effect of allergic fungal sinusitis led to the formation of pseudoaneurysm. Also, the mass effect of polyps and thick secretions in the sphenoid sinus masked its presentation and delayed rupture till after surgery when the mass effect was removed.

Severe epistaxis due to rupture of aneurysmal ICA carries a mortality rate up to 30%–50% [10]. However, its presentation is typically delayed after its formation, with an interval of 3 days to 6 months; 50%–80% present by 3 weeks. Epistaxis may be massive and recurrent if the diagnosis and treatment are delayed [11, 12]. Bleeding is usually characterized by numerous episodes, which become more and more severe over time; though the first hemorrhage may also be fatal in rare instances [13].

The gold standard technique for definite diagnosis of ICA pseudoaneurysm is by angiographic imaging, even though magnetic resonance imaging and three-dimensional CT angiography are performed first [12].

The treatment of choice for ICA aneurysms is endovascular therapy, with many advantages of using detachable balloon and/or coil embolization of the ICA. These techniques are more easily accomplished than surgical clipping or ligation and may be used in areas that are relatively inaccessible by surgical alternatives. Advantages of using balloon occlusion include the ability to continuously monitor the patient’s neurologic status during the procedure, since the embolization is performed under local anesthesia. To further ensure patient safety, test occlusion of the ICA with a nondetachable balloon provides an excellent method for determining adequacy of collateral blood flow to the brain. Endovascular embolization allows occlusion proximal and distal to the pseudoaneurysm, resulting in complete exclusion of the lesion from the arterial circulation. This method is advantageous over surgical ligation of the cervical ICA, which commonly results in recurrent bleeding from collateral flow. Confirmation of complete occlusion of the pseudoaneurysm is readily obtained with a postembolization arteriogram [13].

## Conclusion

In this article we report the first case in the English literature of ICA pseudoaneurysm associated with AFRS. Generally, significant and/or recurrent epistaxis with failure to respond to nasal packing should prompt rapid evaluation with a carotid angiogram even in the absence of a history of head trauma. Prompt diagnosis and therapy of pseudoaneurysms of the ICA are essential in order to prevent morbidity and mortality. Current endovascular embolization technique is considered the standard modality in the management of ICA aneurysm and its rupture.
